# ctDNA-adjusted bTMB as a predictive biomarker for patients with NSCLC treated with PD-(L)1 inhibitors

**DOI:** 10.1186/s12916-022-02360-x

**Published:** 2022-05-05

**Authors:** Wei Nie, Zhi-Jie Wang, Kai Zhang, Bing Li, Yi-Ran Cai, Feng-Cai Wen, Ding Zhang, Yue-Zong Bai, Xue-Yan Zhang, Shu-Yuan Wang, Lei Cheng, Hua Zhong, Li Liu, Jie Wang, Bao-Hui Han

**Affiliations:** 1 Department of Pulmonary Medicine, Shanghai Chest Hospital, Shanghai Jiaotong University, Shanghai, 200030 China; 2 State Key Laboratory of Molecular Oncology, Department of Medical Oncology, National Cancer Center/National Clinical Research Center for Cancer/Cancer Hospital, Chinese Academy of Medical Sciences and Peking Union Medical College, Beijing, China; 3 Cancer Center, Wuhan Union Hospital, Tongji Medical College, Huazhong University of Science and Technology, Wuhan, Hubei China; 4 Department of DataScience, Burning Rock Biotech, Guangzhou, China; 5 The Medical Department, 3D Medicines Inc., Shanghai, China

**Keywords:** ctDNA, Blood tumor mutational burden, NSCLC, Immune checkpoint inhibitor

## Abstract

**Background:**

In non-small cell lung cancer (NSCLC) patients receiving immune checkpoint inhibitors (ICIs), higher blood tumor mutational burden (bTMB) was usually associated with better progression-free survival (PFS) and objective response rate (ORR). However, the association between bTMB and overall survival (OS) benefit remains undefined. It has been reported that patients harboring a high level of circulating tumor DNA (ctDNA) had poor survival. We hypothesized that ctDNA-adjusted bTMB might predict OS benefit in NSCLC patients receiving ICIs.

**Methods:**

Our study was retrospectively performed in three cohorts, including OAK and POPLAR cohort (*n* = 853), Shanghai and Wuhan (SH&WH) cohort (*n* = 44), and National Cancer Center (NCC) cohort (*n* = 47). Durable clinical benefit (DCB) was defined as PFS lasting ≥ 6 months. The cutoff value of ctDNA-adjusted bTMB for DCB prediction was calculated based on a receiver operating characteristic curve. Interaction between treatments and ctDNA-adjusted bTMB was assessed.

**Results:**

The bTMB score was significantly associated with tumor burden, while no association was observed between ctDNA-adjusted bTMB with tumor burden. In the OAK and POPLAR cohort, significantly higher ORR (*P* = 0.020) and DCB (*P* < 0.001) were observed in patients with high ctDNA-adjusted bTMB than those with low ctDNA-adjusted bTMB. Importantly, the interactions between ctDNA-adjusted bTMB and treatments were significant for OS (interaction *P* = 0.019) and PFS (interaction *P* = 0.002). In the SH&WH cohort, the interactions between ctDNA-adjusted bTMB and treatment were marginally significant for OS (interaction *P* = 0.081) and PFS (interaction *P* = 0.062). Similar result was demonstrated in the NCC cohort.

**Conclusions:**

Our study indicated that ctDNA-adjusted bTMB might predict OS benefit in NSCLC patients receiving ICIs. The potential of ctDNA-adjusted bTMB as a noninvasive predictor for immunotherapy should be confirmed in future studies.

**Supplementary Information:**

The online version contains supplementary material available at 10.1186/s12916-022-02360-x.

## Background

Immune checkpoint inhibitors (ICIs) targeting programmed cell death receptor-1 (PD-1) or its ligand (PD-L1) have substantially improved the clinical outcomes of driver-negative non-small-cell lung cancer (NSCLC) patients [1–4]. However, only a minority of patients with NSCLC experienced durable clinical benefit (DCB) from ICIs monotherapy. Although ICIs in combination with chemotherapy showed significantly longer overall survival (OS) and progression-free survival (PFS) than chemotherapy alone, adverse events (AEs) and AE-induced discontinuation of treatment were frequently occurred [5, 6]. Therefore, it is important to develop more appropriate biomarker for clinical effect prediction [7, 8].

Tissue-based tumor mutational burden (tTMB) and PD-L1 expression have been approved as clinical biomarkers of response to ICIs [8, 9]. However, the assessment of tTMB and PD-L1 expression from tissue biopsy samples became challenging in patients with advanced NSCLC, due to inadequate sample quality and quantity, risk of bleeding, spatial and temporal heterogeneity of tumor lesions, and dynamic host immunity. Liquid biopsy showed several advantages than tissue biopsy, such as minimally invasive procedure, easily repeated collection over time, and comprehensive analysis of tumor mutational status and heterogeneity [10].

Recently, assessment of blood-based TMB (bTMB) from circulating tumor DNA (ctDNA) became an attractive method to evaluate clinical benefit of immunotherapy [11–15]. For example, high bTMB was associated with significantly improved objective response rate (ORR) and PFS in NSCLC [12, 13]. Nevertheless, high bTMB could not result in longer OS [12, 16]. In addition, the final result from a prospective phase II trial (B-F1RST) also found that high bTMB did not significantly correlated with treatment benefit for OS [17]. Our previous study in NSCLC patients receiving ICIs observed an upside-down U-shaped curve between bTMB and OS in which both low and high bTMB levels showed better prognosis than patients with medium bTMB level [18]. In the phase III MYSTIC trial, a similar upside-down U-shaped curve between bTMB and OS among NSCLC patients treated with durvalumab was observed (Additional file 1: Fig. S1) [19], indicating that high bTMB was not predictive of OS benefit between patients receiving durvalumab and chemotherapy [20]. Collectively, these results demonstrated that bTMB may not be directly used to predict OS benefit in the setting of ICIs monotherapy [12, 16–20].

ctDNA is released from tumors into bloodstream through apoptosis, necrosis, or active secretion [11, 21]. Avanzini et al. revealed linear correlation between the amount of ctDNA and tumor size, and suggested ctDNA could be a surrogate for tumor burden [22]. Assessment of bTMB in ctDNA relied on total tumor burden [12], which might explain the upside-down U-shaped curve and problems of bTMB predicting OS benefit. Thus, we hypothesized that ctDNA-adjusted bTMB was independent of tumor burden and set out to explore if ctDNA-adjusted bTMB could be a clinically actionable biomarker for prediction of OS in patients with NSCLC receiving ICIs monotherapy.

## Methods

### Study design and patient population

This was a retrospective multicenter study. A total of 944 NSCLC patients from three independent cohorts were included. OAK and POPLAR cohort (*n* = 853) were randomized clinical trials of atezolizumab vs. docetaxel for advanced NSCLC patients failed to platinum-based chemotherapies [3, 4]. The clinical and genetic data were obtained from a previous publication [12]. Ethical review of this cohort was waived by the institutional review board. The Shanghai and Wuhan (SH&WH) cohort included 44 patients with NSCLC from Shanghai Chest Hospital and Wuhan Union Hospital, Tongji Medical College. This study was approved by the Ethics Committee of Shanghai Chest Hospital (Institutional review board No. IS2118) and Wuhan Union Hospital (Institutional review board No. 2017-247). Written informed consent was obtained from patients or their guardians. The National Cancer Center (NCC) cohort included 47 patients with NSCLC from the Cancer Hospital at the Chinese Academy of Medical Sciences, Peking Union Medical College, and Xinqiao Hospital. The study was approved by the Ethics Committee of the NCC (Institutional review board No. NCC2018–092), and all the patients provided written informed consent.

### Evaluation of bTMB and ctDNA-adjusted bTMB

Blood-based targeted next-generation sequencing (NGS) was performed for each patient. The bTMB score was calculated as previously described [12, 13, 16, 23]. In brief, FoundationOne CDx (F1CDx) assay was used through NGS in OAK and POPLAR cohort [12]. NCC-GP150 by 3D Medicines Inc. and OncoScreen Plus by Burning Rock Biotech, Ltd., were used in NCC and WH&SH cohorts, respectively [13, 16, 23]. The gene lists of F1CDx, NCC-GP150, and OncoScreen Plus are shown in Additional file 1: Table. S1. Cell-free DNA (cfDNA) is composed of ctDNA from tumors and circulating DNA from white blood cells, etc. Thus, the mass of ctDNA (ng) cannot be quantified directly. Newman et al. suggested that the level of ctDNA input mass (ng) could be determined as the product of cell-free DNA (cfDNA) input mass and mean allele frequency (AF) of somatic mutations [24]. Therefore, the mass of cfDNA and mean AF of somatic mutations were used to assess ctDNA input mass (ng) in this study. Consequently, ctDNA-adjusted bTMB was calculated as follow:

$$\mathrm{ctDNA}-\mathrm{adjusted}\ \mathrm{bTMB}=\frac{bTMB}{ctDNA\ input\ mass}=\frac{bTMB}{cfDNA\ input\ mass\times mean\ AF\ }$$

### Outcomes

OS was defined as the time from randomization or the initial treatment to death from any cause. PFS and ORR were determined by a clinical radiographic assessment based on the Response Evaluation Criteria in Solid Tumors 1.1. DCB was defined as PFS of 6 months or more, whereas no durable benefit (NDB) was defined as progression of disease within 6 months [25, 26].

### Statistical analysis

Mann–Whitney *U* test or Kruskal-Wallis test were applied to examine the difference between two or more groups. Categorical data were compared using the *χ*^2^ test or Fisher’s exact test, as appropriate. OS and PFS were estimated with the Kaplan-Meier method and log-rank test. The univariate and multivariate Cox proportional hazards model was utilized to estimate the hazard ratios (HRs) and 95% confidence intervals (CIs) for the outcomes.

Receiver operating characteristic (ROC) curves of DCB were plotted by sensitivity and 1-specificity. The area under the curve (AUC) was calculated. The optimal cutoff point was determined by Youden’s index. Restricted cubic spline (RCS) analysis in the Cox proportional hazard model was used to examine the non-linear relationship between a continuous prognostic variable (e.g., bTMB and ctDNA-adjusted bTMB) and an outcome (e.g., HR of PFS or OS) [27].

For all analyses, *P* < 0.05 was considered statistically significant in all 2-tailed tests. The statistical analyses were performed using the R version 3.6.1 (R Project for Statistical Computing) and SPSS version 23.0 (IBM, Armonk, NY).

## Results

### Clinical characteristics of the patient population

**Fig. 1:**
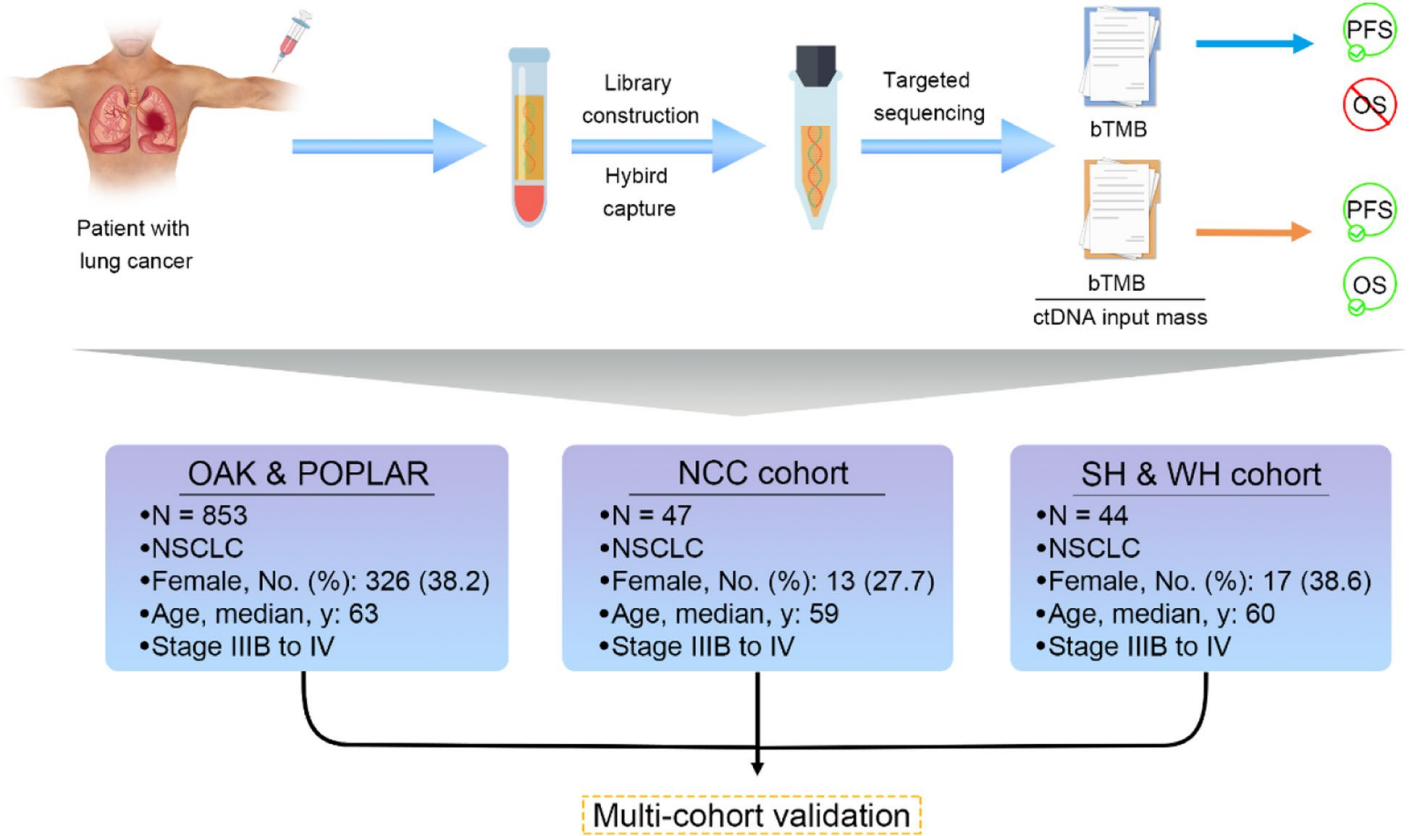
Study schematic. Blood-based next-generation sequencing was performed before NSCLC patients receiving immune checkpoint inhibitors. OAK and POPLAR cohort, National Cancer Center (NCC) cohort, and Shanghai and Wuhan (SH&WH) cohort were used to assess the predictive value of ctDNA-adjusted bTMB

A schematic summary of this study is presented in Fig. 1. The baseline characteristics of OAK and POPLAR cohort, SH&WH cohort, and NCC cohort are shown in Additional file 1: Tables. S2-4. The ctDNA-adjusted bTMB were calculated for each patient. All patients received immunotherapy or chemotherapy.

### The association between ctDNA-adjusted bTMB and tumor burden

There was a small but significant positive spearman correlation between bTMB score and the sum of the longest diameters (Spearman *r* = 0.246, *P* < 0.001, Additional file 1: Fig. S2A) and the number of metastatic sites (*P* < 0.001, Additional file 1: Fig. S2B) in OAK and POPLAR cohort. However, no associations were observed between ctDNA-adjusted bTMB with the sum of longest diameters of target lesions at baseline (Spearman *r* = 0.005, *P* = 0.880, Additional file 1: Fig. S2C) or the number of metastatic sites (*P* = 0.107, Additional file 1: Fig. S2D). These results indicated that ctDNA-adjusted bTMB was independent of tumor burden.

### The predictive role of ctDNA-adjusted bTMB in different cohorts

1. OAK and POPLAR cohort

**Fig. 2:**
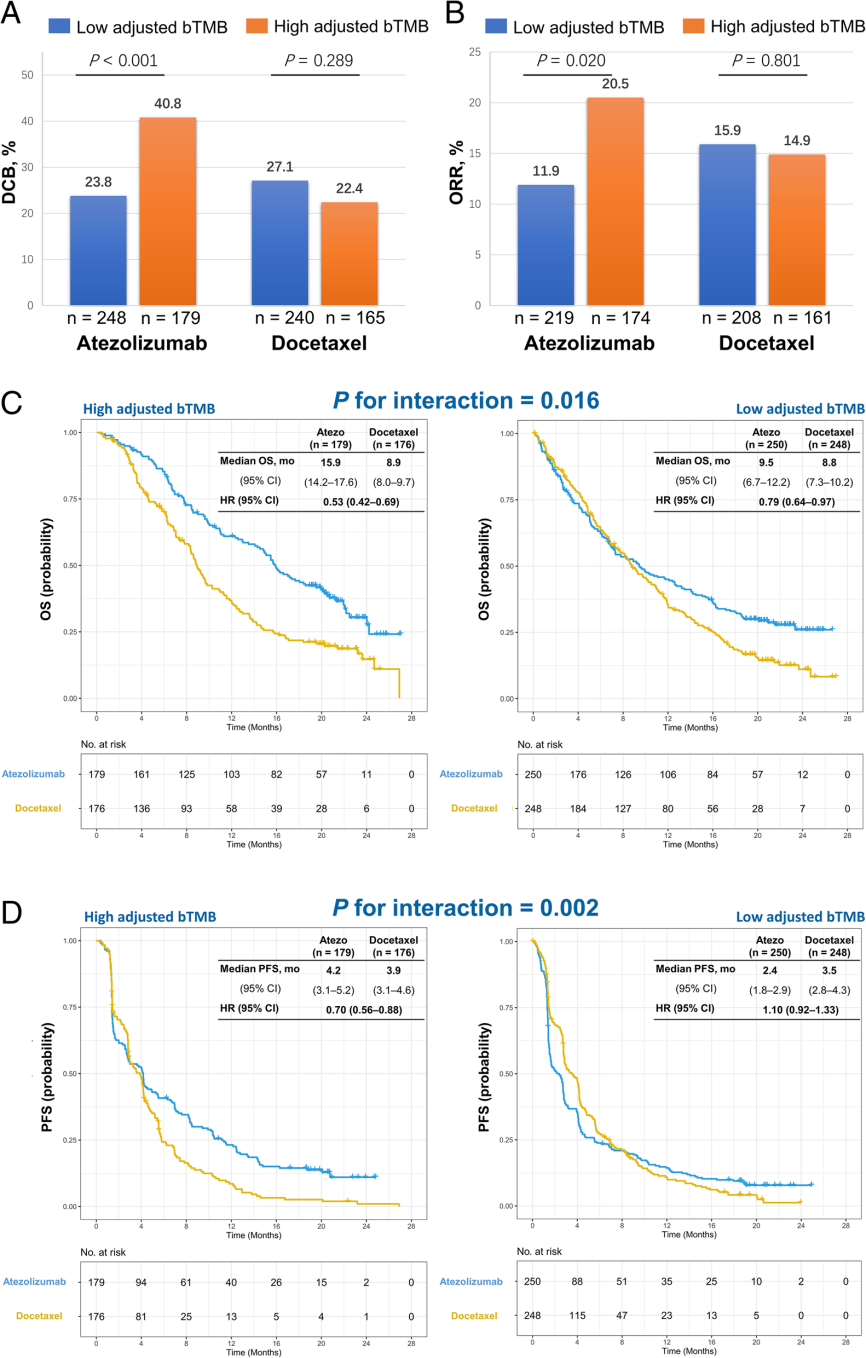
Associations of ctDNA-adjusted bTMB in patients receiving atezolizumab vs. docetaxel treatment. Comparison of (**A**) durable clinical benefit (DCB) and (**B**) objective response rate (ORR) between patients with high and low ctDNA-adjusted bTMB in atezolizumab arm and docetaxel arm. Predictive capacity for (**C**) OS and (**D**) PFS is stratified by treatment with atezolizumab vs. docetaxel in patients with low and high ctDNA-adjusted bTMB in OAK and POPLAR cohort

The genomic mutational landscape and clinical characteristics of patients from OAK and POPLAR cohort are shown in Additional file 1: Fig. S3. The associations between bTMB, ctDNA-adjusted bTMB, and clinical outcomes were assessed. The RCS models showed non-linearly associations between the level of bTMB and HR for PFS and OS (Additional file 1: Fig. S4A left and S4B left). When the bTMB level was adjusted by ctDNA, it was linearly correlated with HR for PFS and OS (Additional file 1: Fig. S4A right and S4B right). The ROC curves were used to indicate the predictive ability of bTMB and ctDNA-adjusted bTMB for DCB. The ctDNA-adjusted bTMB showed better predictive performance than unadjusted bTMB (AUC: 0.63 vs 0.46, *P* = 0.013, Additional file 1: Fig. S5). The optimal cutoff value of ctDNA-adjusted bTMB for predicting DCB was 8 muts/Mb × ng (Additional file 1: Fig. S5). In patients receiving atezolizumab, high ctDNA-adjusted bTMB was significantly associated with improved DCB (*P* < 0.001, Fig. 2A) and ORR (*P* = 0.020, Fig. 2B). However, no significant associations of ctDNA-adjusted bTMB with DCB (*P* = 0.289, Fig. 2A) and ORR (*P* = 0.801, Fig. 2B) were observed in the docetaxel arm. Notably, the interaction *P* values for atezolizumab vs. docetaxel treatment were positive for OS (*P* = 0.016) and PFS (*P* = 0.002), which indicated that high ctDNA-adjusted bTMB might predict better outcomes with ICIs treatment (Fig. 2C and D).

**Fig. 3:**
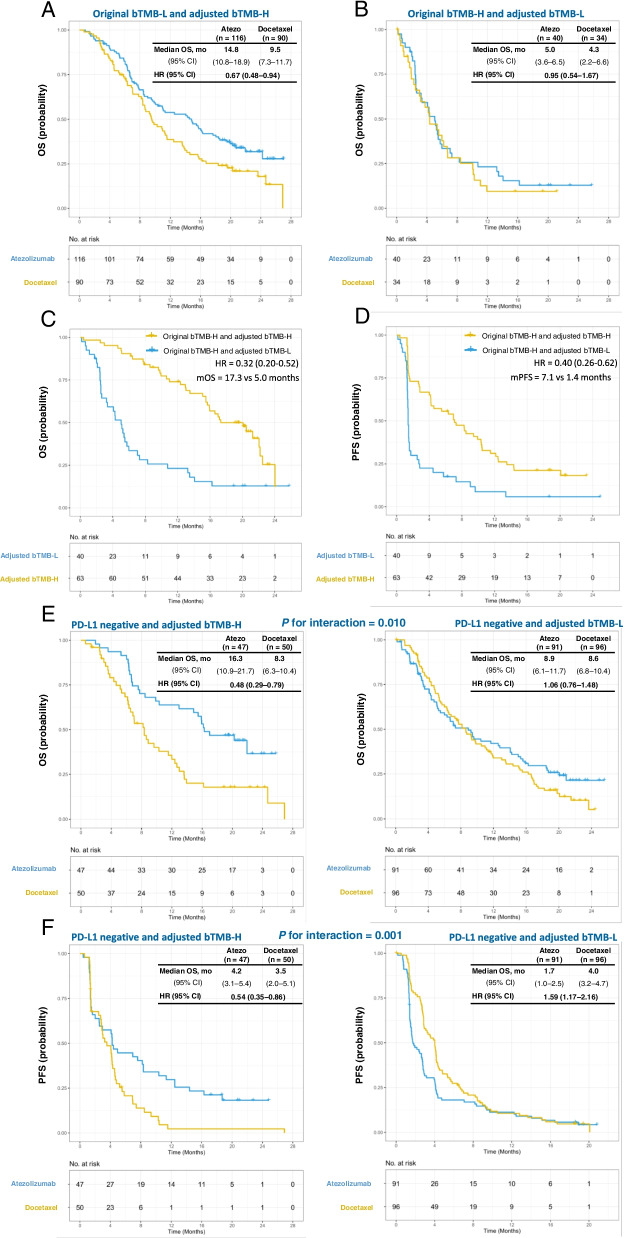
Kaplan–Meier estimates of OS in patients with (**A**) low original bTMB and high ctDNA-adjusted bTMB and (**B**) high original bTMB and low ctDNA-adjusted bTMB, according to treatment group. Kaplan–Meier curves of (**C**) OS and (**D**) PFS in patients with high original bTMB and different levels of ctDNA-adjusted bTMB in atezolizumab arm. Predictive capacity for (**E**) OS and (**F**) PFS is stratified by treatment with atezolizumab vs. docetaxel in patients with PD-L1 negative expression and different levels of ctDNA-adjusted bTMB

In the patients with original low bTMB but high ctDNA-adjusted bTMB, significantly longer median OS was found in treatment arm of atezolizumab than docetaxel (HR = 0.67, 95% CI: 0.48-0.94, Fig. 3A), while the OS in patients with original high bTMB but low ctDNA-adjusted bTMB were comparable between different treatment arms (HR = 0.95, 95% CI: 0.54–1.67, Fig. 3B). Among the patients with atezolizumab treatment and original high bTMB, high ctDNA-adjusted bTMB was significantly associated with improved median OS and median PFS (OS, HR = 0.32, 95% CI: 0.20–0.52, Fig. 3C; PFS, HR = 0.40, 95% CI: 0.26–0.62, Fig. 3D). Then, our study explored the role of ctDNA-adjusted bTMB in patients with negative PD-L1 expression. Indeed, the interactions between ctDNA-adjusted bTMB and treatment arms were significant for OS (interaction *P* = 0.010, Fig. 3E) and PFS (interaction *P* = 0.001, Fig. 3F). Furthermore, we found that ctDNA-adjusted bTMB was predictive for OS in patients with *serine/threonine kinase 11 (STK11)* or *Kelch-like ECH-associated protein 1 (KEAP1)* mutation (Additional file 1: Table S5).

(2) Shanghai and Wuhan (SH&WH) cohort

**Fig. 4:**
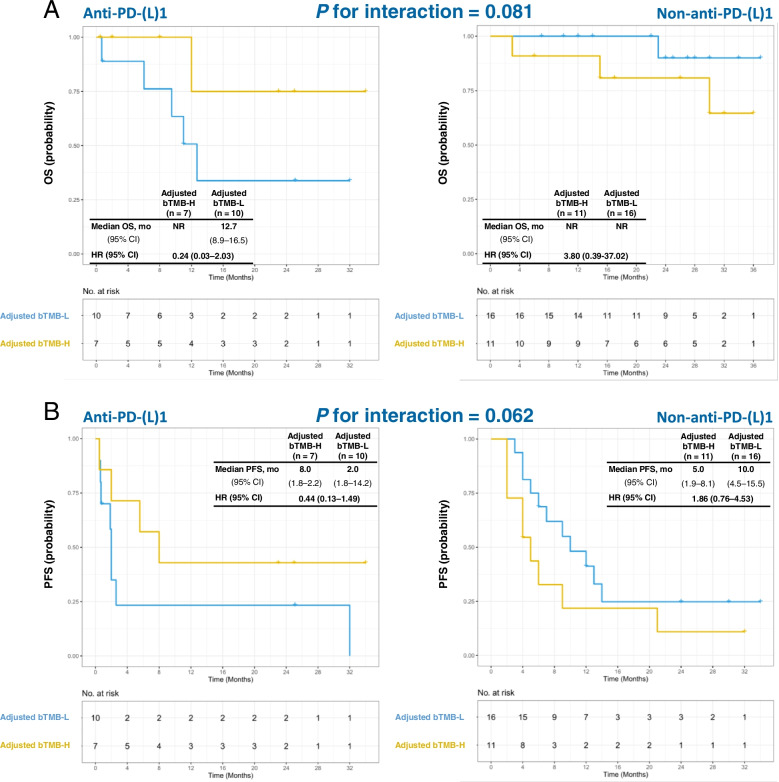
The interactions between ctDNA-adjusted bTMB and treatment in SH&WH cohort. Predictive capacity for (**A**) OS and (**B**) PFS is stratified by high vs. low ctDNA-adjusted bTMB in patients receiving different treatments

The general characteristics and top 20 gene alterations are shown in Additional file 1: Fig. S6. In the ROC curve, an optimal ctDNA-adjusted bTMB cutoff value of 6 muts/Mb × ng was used to obtain the maximum AUC of 0.683, with sensitivity of 66.7% and specificity of 70.0% (Additional file 1: Fig. S7) to predict DCB. The interactions between ctDNA-adjusted bTMB and treatment were marginally significant for OS (interaction *P* = 0.081, Fig. 4A) and PFS (interaction *P* = 0.062, Fig. 4B), suggesting the potential to use ctDNA-adjusted bTMB in predicting treatment benefits of ICIs.

(3) National Cancer Center (NCC) cohort

**Fig. 5:**
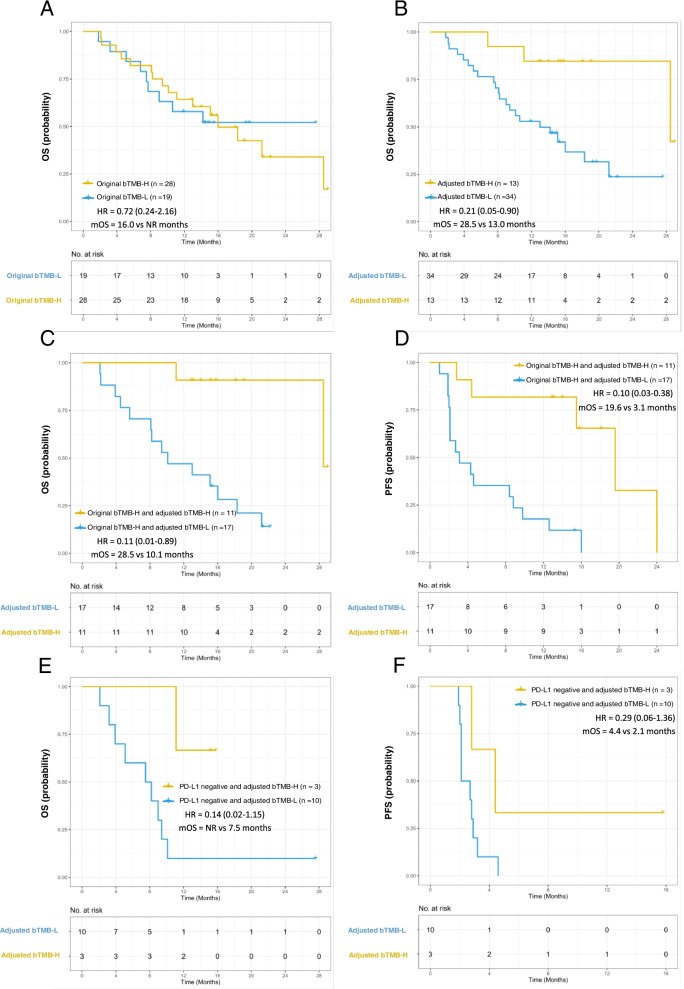
Association between ctDNA-adjusted bTMB and OS or PFS in NCC cohort. **A** Kaplan-Meier survival curve of OS comparing patients treated with immunotherapy with bTMB of less than 6 muts/Mb×ng and bTMB of at least 6 muts/Mb×ng. **B** Kaplan-Meier survival curve of OS comparing patients treated with immunotherapy with ctDNA-adjusted bTMB of less than 11 muts/Mb×ng and bTMB of at least 11 muts/Mb×ng. Kaplan–Meier curves of (**C**) OS and (**D**) PFS in patients with high original bTMB and different levels of ctDNA-adjusted bTMB. Kaplan–Meier curves of (**E**) OS and (**F**) PFS in patients with PD-L1 negative expression and different levels of ctDNA-adjusted bTMB

The clinical and molecular features of NCC cohort are shown in Additional file 1: Fig. S8. We found that bTMB was higher in patients with four or more metastatic sites (*P* = 0.015, Additional file 1: Fig. S9A). No significant association was found between bTMB and OS in NCC cohort (HR = 0.72, 95% CI: 0.24–2.16, Fig. 5A). After ctDNA adjustment, no differences of ctDNA-adjusted bTMB levels were observed between patients with metastatic sites ≥4 and metastatic sites < 4 (*P* = 0.278, Additional file 1: Fig. S9B). The optimized cutoff value of ctDNA-adjusted bTMB for predicting DCB was 11 muts/Mb × ng by the ROC curve (Additional file 1: Fig. S10A). Higher DCB rate and ORR were found in those with ctDNA-adjusted bTMB above versus below 11 muts/Mb × ng (DCB, 61.5% vs. 26.5%, *P* = 0.041, Additional file 1: Fig. S10B; ORR, 46.2% vs. 17.6%, *P* = 0.065, Additional file 1: Fig. S10C). Compared with patients with low ctDNA-adjusted bTMB, patients with high ctDNA-adjusted bTMB demonstrated superior OS (28.5 vs. 13.0 months, HR = 0.21, 95% CI: 0.05–0.90, Fig. 5B) and were more likely to undergo tumor shrinkage (Additional file 1: Fig. S11).

Among patients with original high bTMB, high ctDNA-adjusted bTMB was significantly associated with prolonged median OS and median PFS after ICIs treatment (OS, HR = 0.11, 95% CI: 0.01–0.89, Fig. 5C; PFS, HR = 0.10, 95% CI: 0.03–0.38, Fig. 5D). We further explored the association between ctDNA-adjusted bTMB and clinical outcomes in NSCLC patients with PD-L1 negative expression. In this subgroup, patients with high ctDNA-adjusted bTMB had longer median OS and median PFS than those with low ctDNA-adjusted bTMB (Figs. 5).

## Discussion

In the present study, ctDNA-adjusted bTMB showed superior performance than bTMB in predicting OS for advanced NSCLC patients with ICIs. Compared with non-ICIs treatments, survival advantages of ICIs treatments were observed in patients with high ctDNA-adjusted bTMB.

The use of ctDNA-adjusted bTMB to predict treatment outcomes of ICIs in patients with NSCLC could be explained by two reasons. First, several studies have suggested a strong association between ctDNA levels and tumor burden [21, 28, 29]. We also observed a positive correlation between bTMB score and tumor burden in the OAK and POPLAR cohort as well as the NCC cohort. This correlation, however, did not remain statistically significant after correction for ctDNA level in our study. Second, ctDNA-adjusted bTMB and original bTMB were positively correlated in our study. Thus, tumors with high ctDNA-adjusted bTMB were also likely to produce neoantigens and yielded better response to ICIs.

The use of bTMB and PD-L1 expression as biomarkers for ICIs treatment showed several limitations. Some patients with high bTMB could not benefit from ICIs treatment [18], while some patients with PD-L1 negative expression responded well to ICIs. Thus, there is an urgent need to develop an appropriate biomarker to identify patients whom might have better clinical outcomes during ICIs treatment. Our data suggested that ctDNA-adjusted bTMB may serve as a predictor of clinical benefit to ICIs in patients with high bTMB or PD-L1 negative expression. Taken together, these results demonstrated that integration of ctDNA and PD-L1 expression could identify patients whom were more likely to achieve durable clinical benefit from ICIs treatment.

Somatic mutations in *STK11* and *KEAP1* were related to decreased T cell–inflamed gene expression profile and have been proposed as biomarkers underlying ICIs resistance in patients with NSCLC [30, 31]. In our previous study, we found that combination of high bTMB and PD-L1 expression could predict longer OS in *STK11* or *KEAP1* mutated patients receiving atezolizumab [32]. In the present study, ctDNA-adjusted bTMB was found to be associated with the effectiveness of atezolizumab in *STK11* or *KEAP1* mutated patients. Collectively, our results in the subgroup patients harboring *STK11* or *KEAP1* mutation indicated that assessment of ctDNA-adjusted bTMB was feasible and could identify patients with improved OS from ICIs.

The serial bTMB measurements may reveal more information than single time point measurement. For example, a recent published study suggested that on-treatment blood TMB can be predictors for immunotherapy plus chemotherapy [33]. Combination of baseline and on-treatment bTMB may gain better performance in predicting ICIs treatment. However, there are no available public NGS sequenced cohorts with two arm (ICIs vs chemo) clinical trial and serial blood samples, which makes the exploration not plausible at present. Therefore, future studies are needed to investigate the predictive role of serial ctDNA-adjusted bTMB for ICIs.

There were some limitations in our study. First, as a retrospective study, confounding factors underlying associations between ctDNA-adjusted bTMB and clinical outcomes could be heterogenous. Although the use of ctDNA-adjusted bTMB was validated in different cohorts, further prospective study was warranted in the future. Second, the optimal cutoff value for ctDNA-adjusted bTMB may vary across NGS panels and ICIs. Thus, the role of ctDNA-adjusted bTMB should be determined in different NGS panels and anti-PD-(L)1 inhibitors. Third, the sample size of NCC and SH&WH cohorts was moderate, which might limit the statistical power of conclusions. Finally, a recent investigation indicated that clonal hematopoiesis constituted a pervasive biological phenomenon in cfDNA sequencing approach [34]. Therefore, ctDNA-adjusted bTMB estimation might be influenced by clonal hematopoiesis.

## Conclusions

In summary, our results showed that ctDNA-adjusted bTMB is a promising biomarker to predict OS benefit of ICIs in NSCLC. Further prospective validation of ctDNA-adjusted bTMB as a predictive biomarker for benefit with immunotherapy is warranted.

## Supplementary Information

**Additional file 1: Figure S1.** Cubic spline graph of the HR and 95% CI for the association between bTMB and OS in NSCLC patients treated with durvalumab in MYSTIC trial. **Table S1.** Gene list of NCC-GP150, F1CDxTM, and OncoScreen Plus. **Table S2.** Patient characteristics in OAK and POPLAR cohorts. **Table S3.** Patient characteristics in SH&WH cohort. **Table S4.** Patient characteristics in NCC cohort. **Figure S2.** Correlations between bTMB, sum of the longest diameters and the number of metastatic sites. Correlations between ctDNA adjusted bTMB, sum of the longest diameters, and the number of metastatic sites. **Figure S3.** Oncoprint and clinical characteristics for patients of OAK and POPLAR cohort. **Figure S4.** Cubic spline graph of the HR and 95% CI for the association between bTMB or ctDNA adjusted bTMB and OS or PFS in NSCLC patients treated with atezolizumab OAK and POPLAR cohort. **Figure S5.** ROC curves of bTMB and ctDNA adjusted bTMB to predict DCB in the OAK and POPLAR cohort. **Table S5.** Treatment interaction for OS in *STK11* or *KEAP1* mutated patients. **Figure S6.** Oncoprint and clinical characteristics for patients in Shanghai and Wuhan cohort. **Figure S7.** ROC curve of ctDNA adjusted bTMB to predict DCB in Shanghai and Wuhan cohort. **Figure S8.** Oncoprint and clinical characteristics for patients of National Cancer Center cohort. **Figure S9.** Comparisons of bTMB and ctDNA adjusted bTMB between patients with metastatic site < 4 and metastatic site ≥ 4. **Figure S10.** ROC curve of ctDNA adjusted bTMB to predict DCB in National Cancer Center cohort. Comparisons of DCB and ORR between patients with high and low ctDNA adjusted bTMB. **Figure S11.** Waterfall plot of observed best response from anti–programmed cell death 1 (anti–PD-1) and anti–programmed cell death ligand 1 (anti–PD-L1) checkpoint inhibitors.

## Data Availability

The data and material of OAK and POPLAR study were derived from a previous publication, which was publicly available at https://www.nature.com/articles/s41591-018-0134-3. Other datasets generated and/or analyzed during the current study are not publicly available; however, any reasonable requests for access to available data underlying the results reported in this article will be considered. Such proposals should be submitted to the corresponding author.

## Abbreviations

ICI: Immune checkpoint inhibitor
bTMB: Blood-based tumor mutation burden
PFS: Progression-free survival
OS: Overall survival
ctDNA: Circulating tumor DNA
NSCLC: Non-small cell lung cancer
DCB: Durable clinical benefit
NDB: No durable benefit
NGS: Next-generation sequencing
PD-L1: Programmed death-ligand 1
ORR: Objective response rate
DCR: Disease control rate
cfDNA: Cell-free DNA
AUC: Area under the curve
RCS: Restricted cubic spline
