# Supplementary material for: ctDNA-adjusted bTMB as a predictive biomarker for patients with NSCLC treated with PD-(L)1 inhibitors
Source: BMC Med. 2022 May 5;20:170. doi: 10.1186/s12916-022-02360-x (PMC9069852; doi:10.1186/s12916-022-02360-x)
Supplement: Supplementary file 1 — Additional file 1: Figure S1. Cubic spline graph of the HR and 95% CI for the association between bTMB and OS in NSCLC patients treated with durvalumab in MYSTIC trial. Table S1. Gene list of NCC-GP150, F1CDxTM, and OncoScreen Plus. Table S2. Patient characteristics in OAK and POPLAR cohorts. Table S3. Patient characteristics in SH&WH cohort. Table S4. Patient characteristics in NCC cohort. Figure S2. Correlations between bTMB, sum of the longest diameters and the number of metastatic sites. Correlations between ctDNA adjusted bTMB, sum of the longest diameters, and the number of metastatic sites. Figure S3. Oncoprint and clinical characteristics for patients of OAK and POPLAR cohort. Figure S4. Cubic spline graph of the HR and 95% CI for the association between bTMB or ctDNA adjusted bTMB and OS or PFS in NSCLC patients treated with atezolizumab OAK and POPLAR cohort. Figure S5. ROC curves of bTMB and ctDNA adjusted bTMB to predict DCB in the OAK and POPLAR cohort. Table S5. Treatment interaction for OS in STK11 or KEAP1 mutated patients. Figure S6. Oncoprint and clinical characteristics for patients in Shanghai and Wuhan cohort. Figure S7. ROC curve of ctDNA adjusted bTMB to predict DCB in Shanghai and Wuhan cohort. Figure S8. Oncoprint and clinical characteristics for patients of National Cancer Center cohort. Figure S9. Comparisons of bTMB and ctDNA adjusted bTMB between patients with metastatic site < 4 and metastatic site ≥ 4. Figure S10. ROC curve of ctDNA adjusted bTMB to predict DCB in National Cancer Center cohort. Comparisons of DCB and ORR between patients with high and low ctDNA adjusted bTMB. Figure S11. Waterfall plot of observed best response from anti–programmed cell death 1 (anti–PD-1) and anti–programmed cell death ligand 1 (anti–PD-L1) checkpoint inhibitors. [file 12916_2022_2360_MOESM1_ESM.docx]

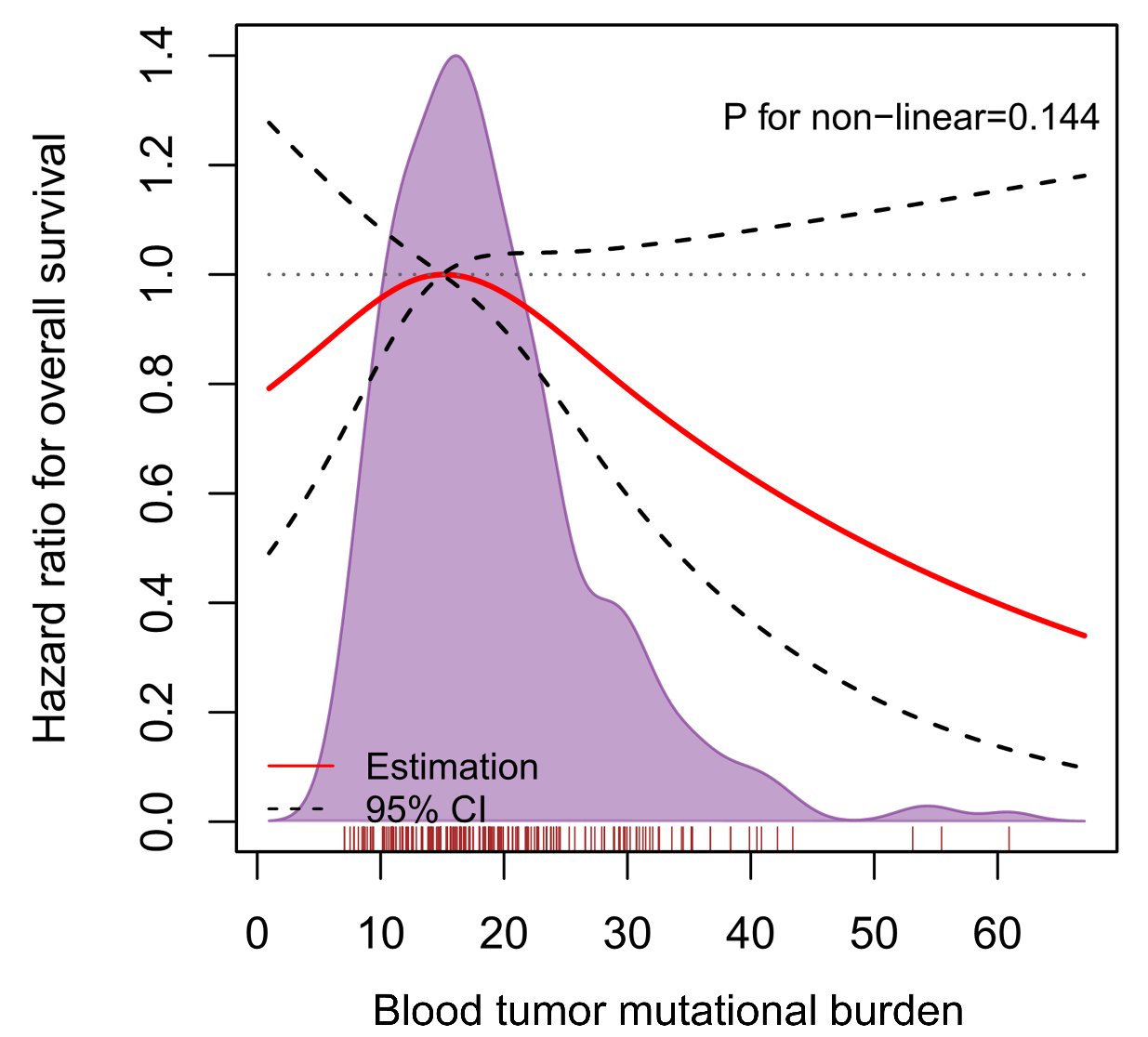


**Figure S1.** Cubic spline graph of the HR (represented by solid red line) and 95% CI (represented by the black dotted lines) for the association between bTMB and OS in NSCLC patients treated with durvalumab in MYSTIC trial. The purple area and brown area indicate the distribution of bTMB.

**Table S1. Gene list of NCC-GP150, F1CDx^TM^, and OncoScreen Plus.**

| **Gene list: NCC-GP150** |
| --- |
| ACVR2A AKT1 AKT2 ALK APC AR ARAF ARID1A ARID2 ATM ATR AXIN1 BARD1 BCL2L11 BIRC5 BRAF BRCA1 BRCA2 BRIP1 C11orf30 CBL CCND1 CCND2 CCNE1 CD274 CDH1 CDK12 CDK4 CDK6 CDKN1B CDKN2A CHEK1 CHEK2 CREBBP CRKL CTNNB1 CYP2C19 CYP2D6 DDR2 DPYD EGFR EP300 EPHB1 ERBB2 ERBB3 ERBB4 ERRFI1 ESR1 EZH2 FAM135B FAT1 FBXW7 FGF19 FGFR1 FGFR2 FGFR3 FLT1 FLT3 FLT4 GATA3 GLI3 GNA11 GNAQ GNAS HNF1A HRAS IDH1 IDH2 IRS2 JAK2 JAK3 KDR KEAP1 KIT KMT2A KRAS LRP1B MAP2K1 MAP2K2 MAP2K4 MAP3K1 MCL1 MET MLH1 MRE11A MSH2 MSH6 MTOR MYC MYCL MYCN NF1 NFE2L2 NKX2-1 NOTCH1 NOTCH2 NOTCH3 NRAS NRG1 NRG3 NTRK1 NTRK2 NTRK3 PALB2 PDCD1LG2 PDGFRA PDGFRB PIK3CA PIK3R1 PREX2 PTCH1 PTEN PTK2 PTPN11 RAD50 RAF1 RB1 RBM10 RET RICTOR RIT1 RNF43 ROS1 RUNX1T1 SETD2 SLIT2 SMAD2 SMAD3 SMAD4 SMARCA2 SMARCA4 SMO SOX2 SPEN SPTA1 SRC STK11 TBX3 TCF7L2 TERT TGFBR2 TP53 TPMT TSC1 TSC2 UGT1A1 VEGFA VHL ZNF217 ZNF703 |
| **Gene list: F1CDx^TM^** |
| ABL1 BRAF CDKN1A EPHA3 FGFR4 IKZF1 MCL1 NKX2-1 PMS2 RNF43 TET2 ACVR1B BRCA1 CDKN1B EPHB1 FH INPP4B MDM2 NOTCH1 POLD1 ROS1 TGFBR2 AKT1 BRCA2 CDKN2A EPHB4 FLCN IRF2 MDM4 NOTCH2 POLE RPTOR TIPARP AKT2 BRD4 CDKN2B ERBB2 FLT1 IRF4 MED12 NOTCH3 PPARG SDHA TNFAIP3 AKT3 BRIP1 CDKN2C ERBB3 FLT3 IRS2 MEF2B NPM1 PPP2R1A SDHB TNFRSF14 ALK BTG1 CEBPA ERBB4 FOXL2 JAK1 MEN1 NRAS PPP2R2A SDHC TP53 ALOX12B BTG2 CHEK1 ERCC4 FUBP1 JAK2 MERTK NT5C2 PRDM1 SDHD TSC1 AMER1 BTK CHEK2 ERG GABRA6 JAK3 MET NTRK1 PRKAR1A SETD2 TSC2 APC C11orf30 CIC ERRFI1 GATA3 JUN MITF NTRK2 PRKCI SF3B1 TYRO3 AR CALR CREBBP ESR1 GATA4 KDM5A MKNK1 NTRK3 PTCH1 SGK1 U2AF1 ARAF CARD11 CRKL EZH2 GATA6 KDM5C MLH1 P2RY8 PTEN SMAD2 VEGFA ARFRP1 CASP8 CSF1R FAM46C GID4 KDM6A MPL PALB2 PTPN11 SMAD4 VHL ARID1A CBFB CSF3R FANCA GNA11 KDR MRE11A PARK2 PTPRO SMARCA4 WHSC1 ASXL1 CBL CTCF FANCC GNA13 KEAP1 MSH2 PARP1 QKI SMARCB1 WHSC1L1 ATM CCND1 CTNNA1 FANCG GNAQ KEL MSH3 PARP2 RAC1 SMO WT1 ATR CCND2 CTNNB1 FANCL GNAS KIT MSH6 PARP3 RAD21 SNCAIP XPO1 ATRX CCND3 CUL3 FAS GRM3 KLHL6 MST1R PAX5 RAD51 SOCS1 XRCC2 AURKA CCNE1 CUL4A FBXW7 GSK3B KMT2A MTAP PBRM1 RAD51B SOX2 ZNF217 AURKB CD22 CXCR4 FGF10 H3F3A KMT2D MTOR PDCD1 RAD51C SOX9 ZNF703 AXIN1 CD274 CYP17A1 FGF12 HDAC1 KRAS MUTYH PDCD1LG2 RAD51D SPEN AXL CD70 DAXX FGF14 HGF LTK MYC PDGFRA RAD52 SPOP BAP1 CD79A DDR1 FGF19 HNF1A LYN MYCL PDGFRB RAD54L SRC BARD1 CD79B DDR2 FGF23 HRAS MAF MYCN PDK1 RAF1 STAG2 BCL2 CDC73 DIS3 FGF3 HSD3B1 MAP2K1 MYD88 PIK3C2B RARA STAT3 BCL2L1 CDH1 DNMT3A FGF4 ID3 MAP2K2 NBN PIK3C2G RB1 STK11 BCL2L2 CDK12 DOT1L FGF6 IDH1 MAP2K4 NF1 PIK3CA RBM10 SUFU BCL6 CDK4 EED FGFR1 IDH2 MAP3K1 NF2 PIK3CB REL SYK BCOR CDK6 EGFR FGFR2 IGF1R MAP3K13 NFE2L2 PIK3R1 RET TBX3 BCORL1 CDK8 EP300 FGFR3 IKBKE MAPK1 NFKBIA PIM1 RICTOR TEK ETV4 EZR NUTM1 SLC34A2 ETV5 TERC BCR CD74 ETV6 RSPO2 TERT EWSR1 MYB SDC4 TMPRSS2 |
| **Gene list: OncoScreen Plus^TM^** |
| ABL1 ABL2 ABRAXAS1 ACVR1 ACVR1B AKT1 AKT2 AKT3 ALK ALOX12B AMER1 APC AR ARAF ARFRP1 ARID1A ARID1B ARID2 ARID5B ASXL1 ASXL2 ATM ATR ATRX AURKA AURKB AXIN1 AXIN2 AXL B2M BAP1 BARD1 BBC3 BCL10 BCL2 BCL2L1 BCL2L11 BCL2L2 BCL6 BCOR BCORL1 BIRC3 BLM BMPR1A BRAF BRCA1 BRCA2 BRD4 BRD7 BRINP3 BRIP1 BTG1 BTG2 BTK CALR CARD11 CASP8 CBFB CBL CCND1 CCND2 CCND3 CCNE1 CD274 CD74 CD79A CD79B CDC73 CDH1 CDH18 CDK12 CDK4 CDK6 CDK8 CDKN1A CDKN1B CDKN1C CDKN2A CDKN2B CDKN2C CEBPA CENPA CHD1 CHD2 CHD4 CHEK1 CHEK2 CIC CREBBP CRKL CRLF2 CSF1R CSF3R CSMD1 CSMD3 CTCF CTLA4 CTNNA1 CTNNB1 CUL3 CUL4A CXCR4 CYLD CYP17A1 CYP2D DAXX DCUN1D1 DDR1 DDR2 DICER1 DIS3 DNAJB1 DNMT1 DNMT3A DNMT3B DOT1L DPYD EED EGFR EIF1AX EIF4E EMSY EP300 EPCAM EPHA2 EPHA3 EPHA5 EPHA7 EPHB1 EPHB4 ERBB2 ERBB3 ERBB4 ERCC1 ERCC2 ERCC3 ERCC4 ERCC5 ERG ERRFI1 ESR1 ETV4 ETV5 ETV6 EWSR1 EZH2 EZR FANCA FANCC FANCD2 FANCE FANCF FANCG FANCI FANCL FANCM FAS FAT1 FBXW7 FGF10 FGF12 FGF14 FGF19 FGF23 FGF3 FGF4 FGF6 FGF7 FGFR1 FGFR2 FGFR3 FGFR4 FH FLCN FLT1 FLT3 FLT4 FOXA1 FOXL2 FOXO1 FOXP1 FRS2 FUBP1 FYN GABRA6 GATA1 GATA2 GATA3 GATA4 GATA6 GEN1 GID4 GLI1 GNA11 GNA13 GNAQ GNAS GPS2 GREM1 GRIN2A GRM3 GSK3B H3F3A H3F3B H3F3C HDAC1 HDAC2 HGF HIST1H1C HIST1H2BD HIST1H3A HIST1H3B HIST1H3C HIST1H3D HIST1H3E HIST1H3G HIST1H3H HIST1H3I HIST1H3J HIST2H3D HIST3H3 HLA-A HLA-B HLA-C HNF1A HOXB13 HRAS HSD3B1 HSP90AA1 ICOSLG ID3 IDH1 IDH2 IFNGR1 IGF1 IGF1R IGF2 IKBKE IKZF1 IL10 IL7R INHA INHBA INPP4A INPP4B INSR IRF2 IRF4 IRS1 IRS2 JAK1 JAK2 JAK3 JUN KAT6A KDM5A KDM5C KDM6A KDR KEAP1 KEL KIT KLF4 KLHL6 KMT2A KMT2C KMT2D KRAS LATS1 LATS2 LMO1 LRP1B LTK LYN MAF MAGI2 MALT1 MAP2K1 MAP2K2 MAP2K4 MAP3K1 MAP3K13 MAPK1 MAPK3 MAX MCL1 MDC1 MDM2 MDM4 MED12 MEF2B MEN1 MERTK MET MGA MIR21 MITF MKNK1 MLH1 MLH3 MPL MRE11 MSH2 MSH3 MSH6 MST1 MST1R MTAP MTOR MUTYH MYC MYCL MYCN MYD88 MYOD1 NAV3 NBN NCOA3 NCOR1 NCOR2 NEGR1 NF1 NF2 NFE2L2 NFKBIA NKX2-1 NKX3-1 NOTCH1 NOTCH2 NOTCH3 NOTCH4 NPM1 NRAS NRG1 NSD1 NSD2 NSD3 NT5C2 NTHL1 NTRK1 NTRK2 NTRK3 NUP93 NUTM1 P2RY8 PAK1 PAK3 PAK5 PALB2 PARP1 PARP2 PARP3 PAX5 PBRM1 PCDH11X PDCD1 PDCD1LG2 PDGFRA PDGFRB PDK1 PGR PHOX2B PIK3C2B PIK3C2G PIK3C3 PIK3CA PIK3CB PIK3CD PIK3CG PIK3R1 PIK3R2 PIK3R3 PIM1 PLCG2 PLK2 PMS1 PMS2 PNRC1 POLD1 POLE PPARG PPM1D PPP2R1A PPP2R2A PPP6C PRDM1 PREX2 PRKAR1A PRKCI PRKDC PRKN PTCH1 PTEN PTPN11 PTPRD PTPRO PTPRS PTPRT QKI RAB35 RAC1 RAD21 RAD50 RAD51 RAD51B RAD51C RAD51D RAD52 RAD54L RAF1 RARA RASA1 RB1 RBM10 RECQL4 REL RET RHEB RHOA RICTOR RIT1 RNF43 ROS1 RPA1 RPS6KA4 RPS6KB2 RPTOR RSPO2 RUNX1 RUNX1T1 SDC4 SDHA SDHAF2 SDHB SDHC SDHD SETD2 SF3B1 SGK1 SH2B3 SH2D1A SHQ1 SLC34A2 SLIT2 SLX4 SMAD2 SMAD3 SMAD4 SMARCA4 SMARCB1 SMARCD1 SMO SNCAIP SOCS1 SOX10 SOX17 SOX2 SOX9 SPEN SPOP SPTA1 SRC SRSF2 STAG2 STAT3 STAT4 STAT5A STAT5B STK11 STK40 SUFU SYK TAF1 TBX3 TCF3 TCF7L2 TEK TENT5C TERC TERT TET1 TET2 TGFBR1 TGFBR2 TIPARP TMEM127 TMPRSS2 TNFAIP3 TNFRSF14 TOP1 TOP2A TP53 TP63 TRAF2 TRAF7 TRIM58 TRPC5 TSC1 TSC2 TSHR TYRO3 U2AF1 UGT1A1 VEGFA VEGFB VHL WISP3 WRN WT1 XIAP XPO1 XRCC2 XRCC3 YAP1 YES1 ZBTB16 ZBTB2 ZNF217 ZNF703 ZNRF3 |

**Table S2.** Patient characteristics in OAK and POPLAR cohorts

| Characteristics | Total (n = 853) |
| --- | --- |
| Age (years), median (range) | 63 (34-85) |
| Race (%) |  |
| White | 616 (72.2) |
| Non-white | 237 (27.8) |
| Gender (% female) | 326 (38.2) |
| Smoking status (%) |  |
| Never | 272 (31.9) |
| Former/current | 581 (68.1) |
| ECOG PS (%) |  |
| 0 | 284 (33.3) |
| 1 | 569 (66.7) |
| Histology (%) |  |
| Non-squamous | 598 (70.1) |
| Squamous | 255 (29.9) |
| Line of therapy (% two) | 627 (73.5) |
| Baseline SLD (mm), median (range) | 73 (10-316) |
| Metastatic site, median (range) | 3 (0-8) |
| PD-L1 (% positive) * | 353 (41.4) |
| PD-L1 (% strong) * | 111 (13.0) |
| bTMB (muts/Mb), median (range) | 8 (0-89) |
| Treatments (%) |  |
| Atezolizumab | 429 (50.3) |
| Docetaxel | 424 (49.7) |

ECOG, Eastern Cooperative Oncology Group; PS, performance status; SLD, sum of the longest diameters; PD-L1, programmed death-ligand 1; bTMB, blood tumor mutational burden.

*Only 637 patients had PD-L1 data.

**Table S3.** Patient characteristics in SH&WH cohort.

| Characteristics | Total (n = 44) |
| --- | --- |
| Age (years), median (range) | 60 (29-77) |
| Gender (% female) | 17 (38.6) |
| Histology (%) |  |
| Non-squamous | 35 (79.5) |
| Squamous | 9 (20.5) |
| Line of therapy (%) |  |
| 1 | 25 (58.1) |
| ≥ 2 | 18 (41.9) |
| Smoking status (%) |  |
| Never | 17 (50.0) |
| Former/current | 17 (50.0) |
| bTMB (muts/Mb), median (range) | 3.6 (0-28.6) |
| Treatments (%) |  |
| Anti-PD-(L)1 | 17 (38.6) |
| Non-anti-PD-(L)1 | 27 (61.4) |

ECOG, Eastern Cooperative Oncology Group; PS, performance status; bTMB, blood tumor mutational burden; PD-L1, programmed death-ligand 1

**Table S4.** Patient characteristics in NCC cohort.

| Characteristics | Total (n = 47) |
| --- | --- |
| Age (years), median (range) | 59 (36-72) |
| Gender (% female) | 13 (27.7) |
| ECOG PS (%) |  |
| 0-1 | 40 (85.1) |
| ≥ 2 | 7 (14.9) |
| Histology (%) |  |
| Non-squamous | 29 (61.7) |
| Squamous | 18 (38.3) |
| Line of therapy (%) |  |
| 1 | 11 (23.4) |
| ≥ 2 | 36 (76.6) |
| Smoking status (%) |  |
| Never | 26 (55.3) |
| Former/current | 21 (44.7) |
| No. of metastatic sites (%) |  |
| < 4 | 31 (66.0) |
| ≥ 4 | 16 (34.0) |
| PD-L1 (% positive) | 22 (46.8) |
| bTMB (muts/Mb), median (range) | 7 (3-19) |
| Treatments (%) |  |
| Anti-PD-1 | 39 (83.0) |
| Anti-PD-L1 | 8 (17.0) |

ECOG, Eastern Cooperative Oncology Group; PS, performance status; PD-L1, programmed death-ligand 1; bTMB, blood tumor mutational burden; PD-1, programmed death 1


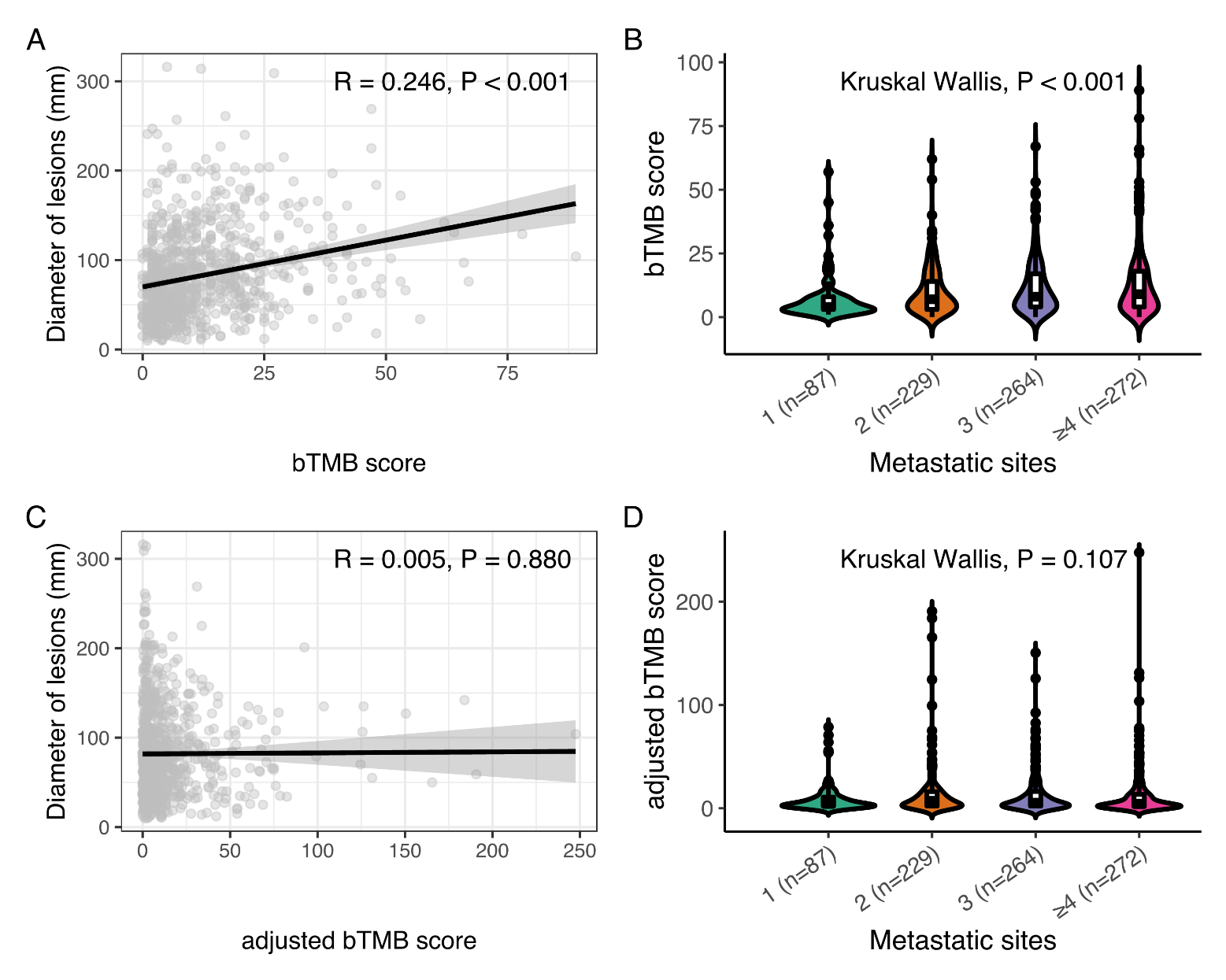


**Figure S2.** Correlations between bTMB, sum of the longest diameters (A), and the number of metastatic sites (B). Correlations between ctDNA adjusted bTMB, sum of the longest diameters (A), and the number of metastatic sites (B).


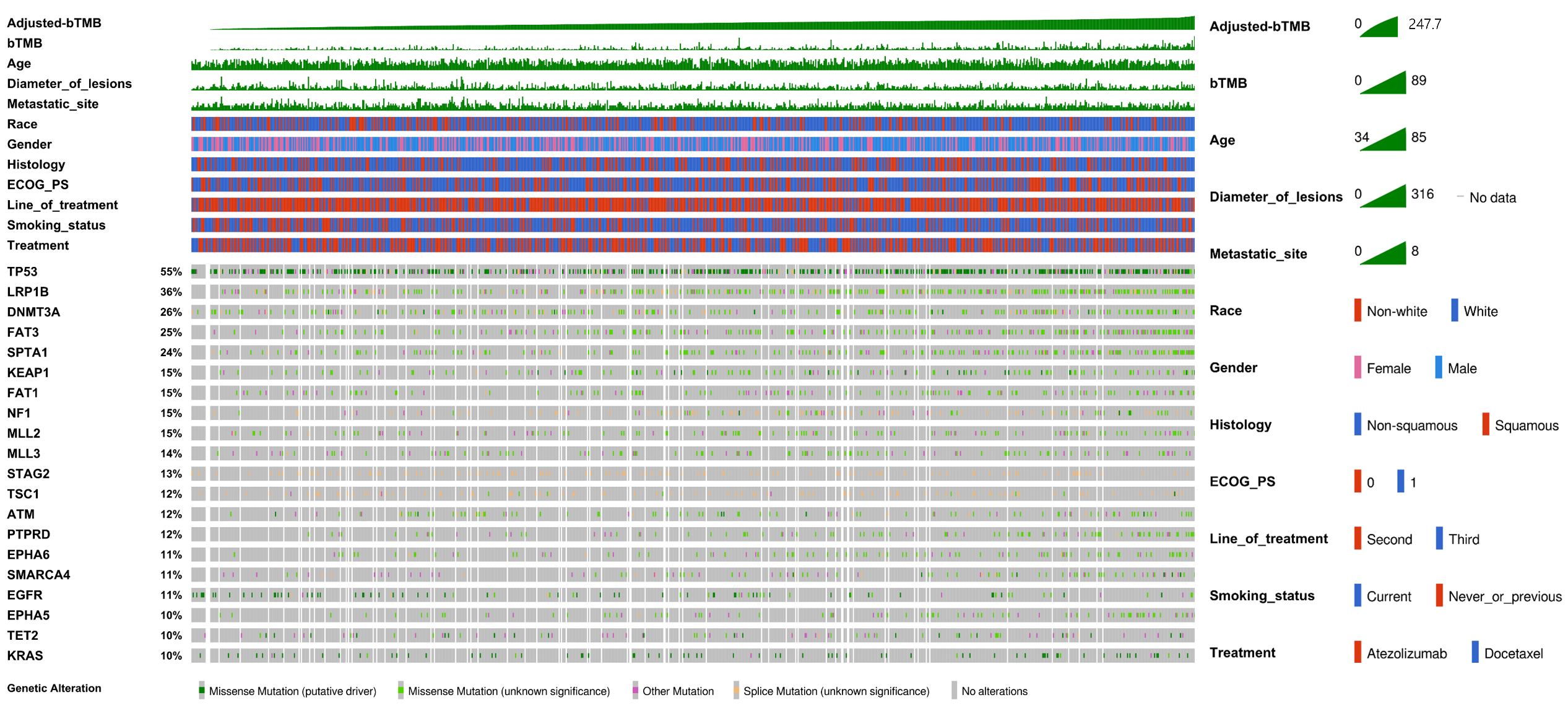


**Figure S3.** Oncoprint and clinical characteristics for patients of OAK and POPLAR cohort.


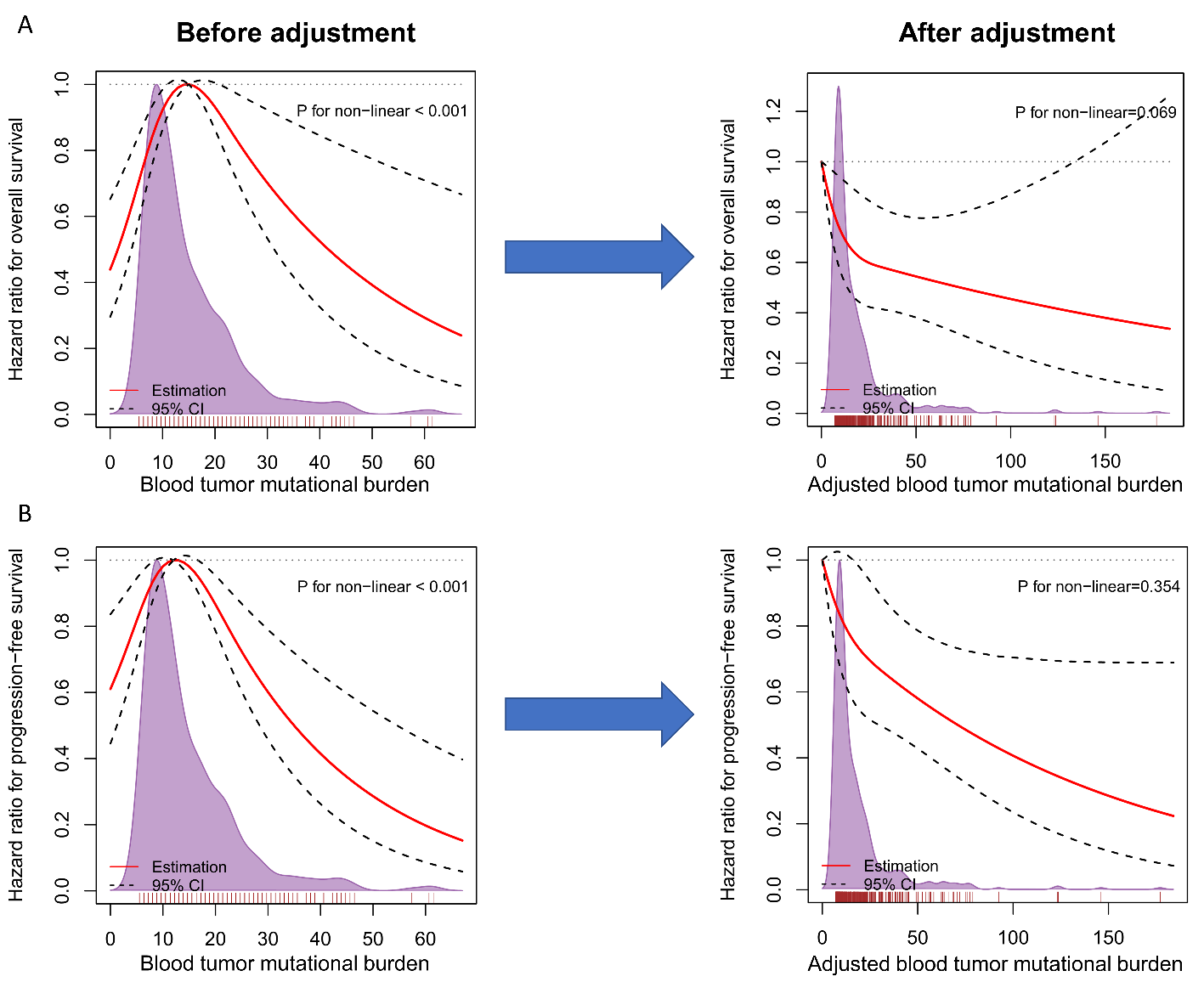


**Figure S4.** Cubic spline graph of the HR (represented by solid red line) and 95% CI (represented by the black dotted lines) for the association between bTMB or ctDNA adjusted bTMB and OS (A) or PFS (B) in NSCLC patients treated with atezolizumab OAK and POPLAR cohort. The purple area and brown area indicate the distribution of bTMB.


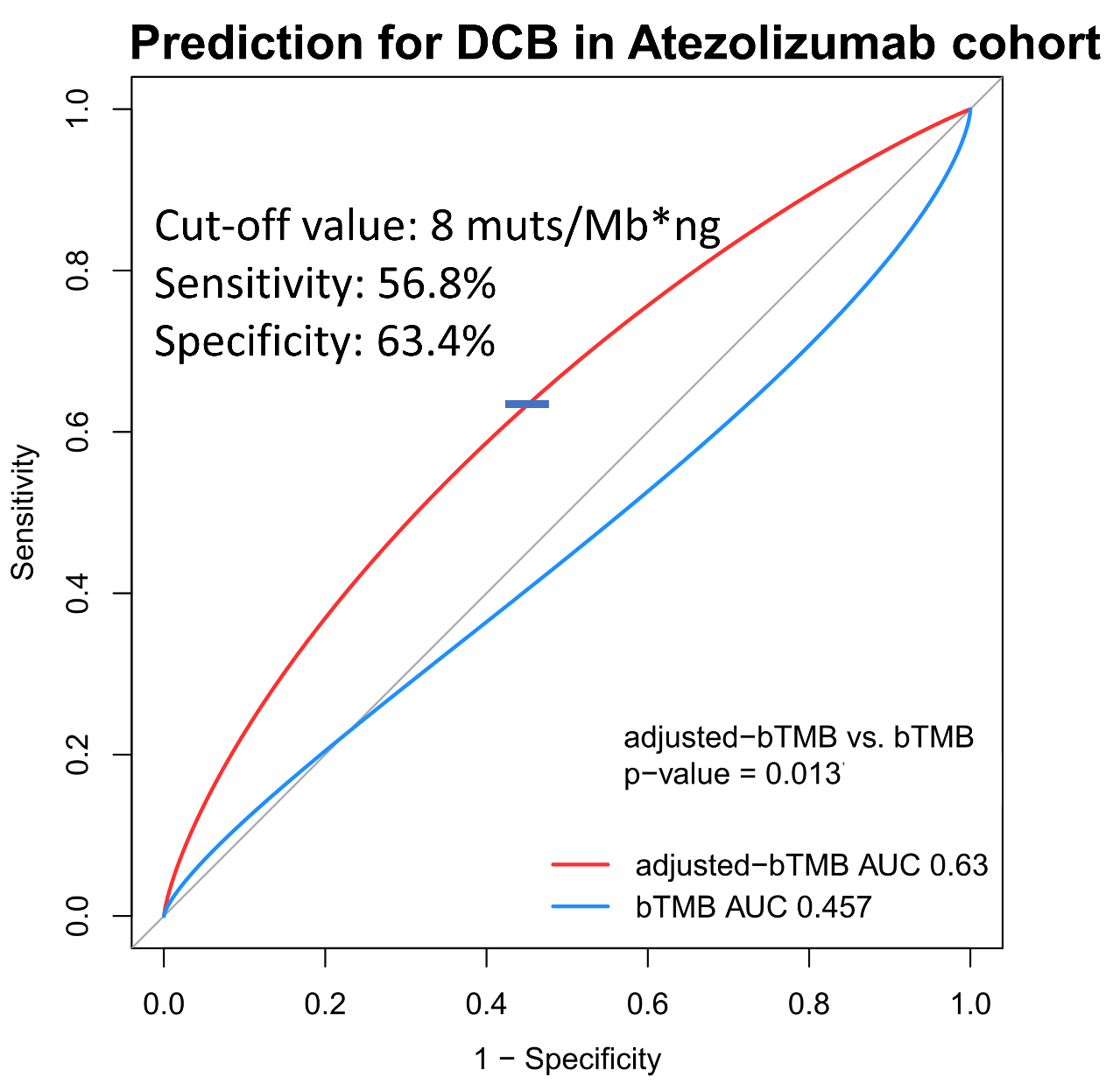


**Figure S5.** ROC curves of bTMB and ctDNA adjusted bTMB to predict DCB in the OAK and POPLAR cohort.

**Table S5.** Treatment interaction for OS in *STK11* or *KEAP1* mutated patients.

|  | Atezolizumab vs. Docetaxel  HR (95%CI) | *P* interaction |
| --- | --- | --- |
| bTMB |  |  |
| < 16 muts/Mb | 0.63 (0.39-1.02) | 0.896 |
| ≥ 16 muts/Mb | 0.66 (0.40-1.10) |  |
| ctDNA adjusted bTMB |  |  |
| < 8 muts/Mb*ng | 1.01 (0.61-1.69) | 0.028 |
| ≥ 8 muts/Mb*ng | 0.45 (0.27-0.75) |  |

OS, overall survival; HR, hazard ratio; CI, confidence interval.


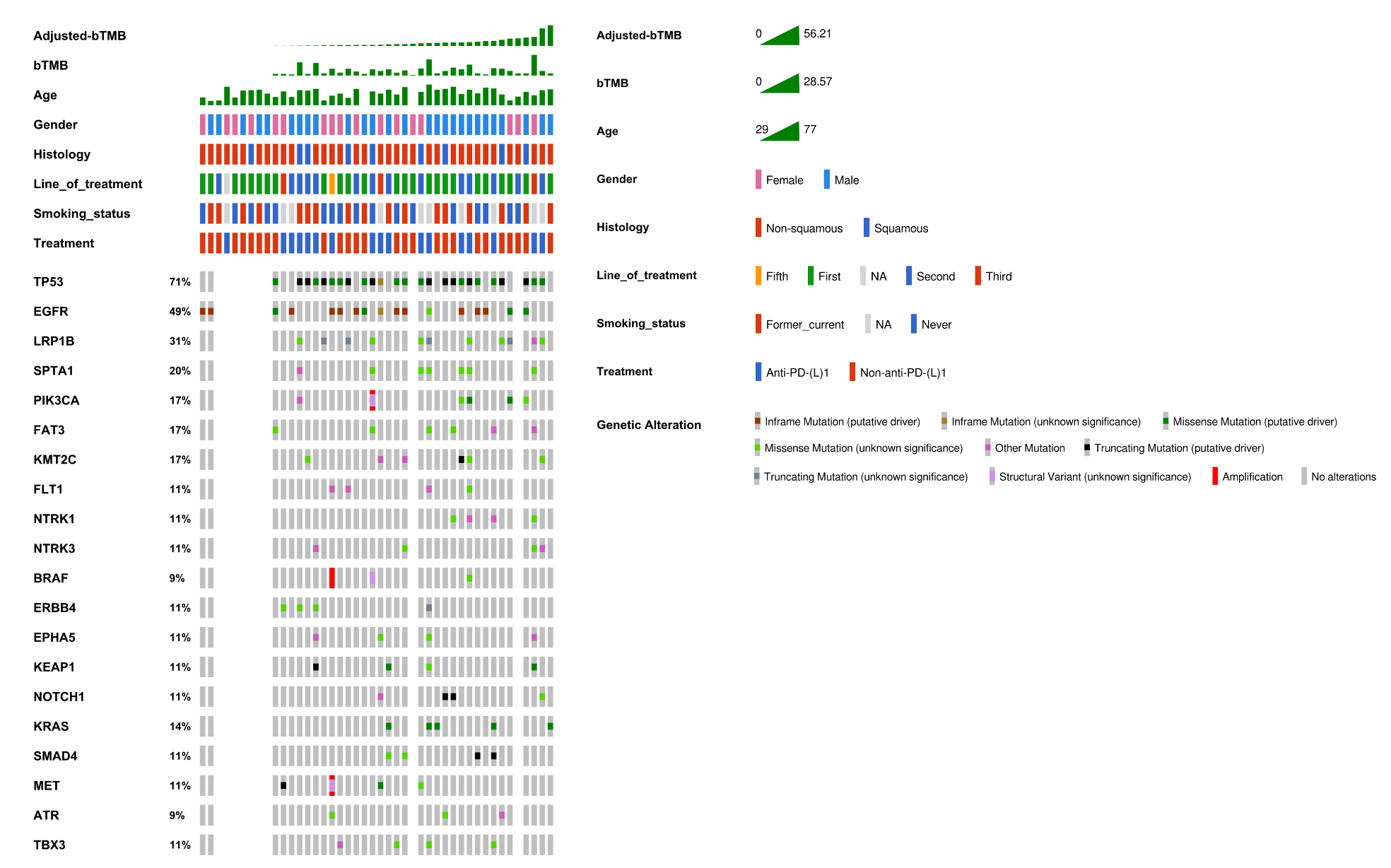


**Figure S6.** Oncoprint and clinical characteristics for patients in Shanghai and Wuhan (SH&WH) cohort.


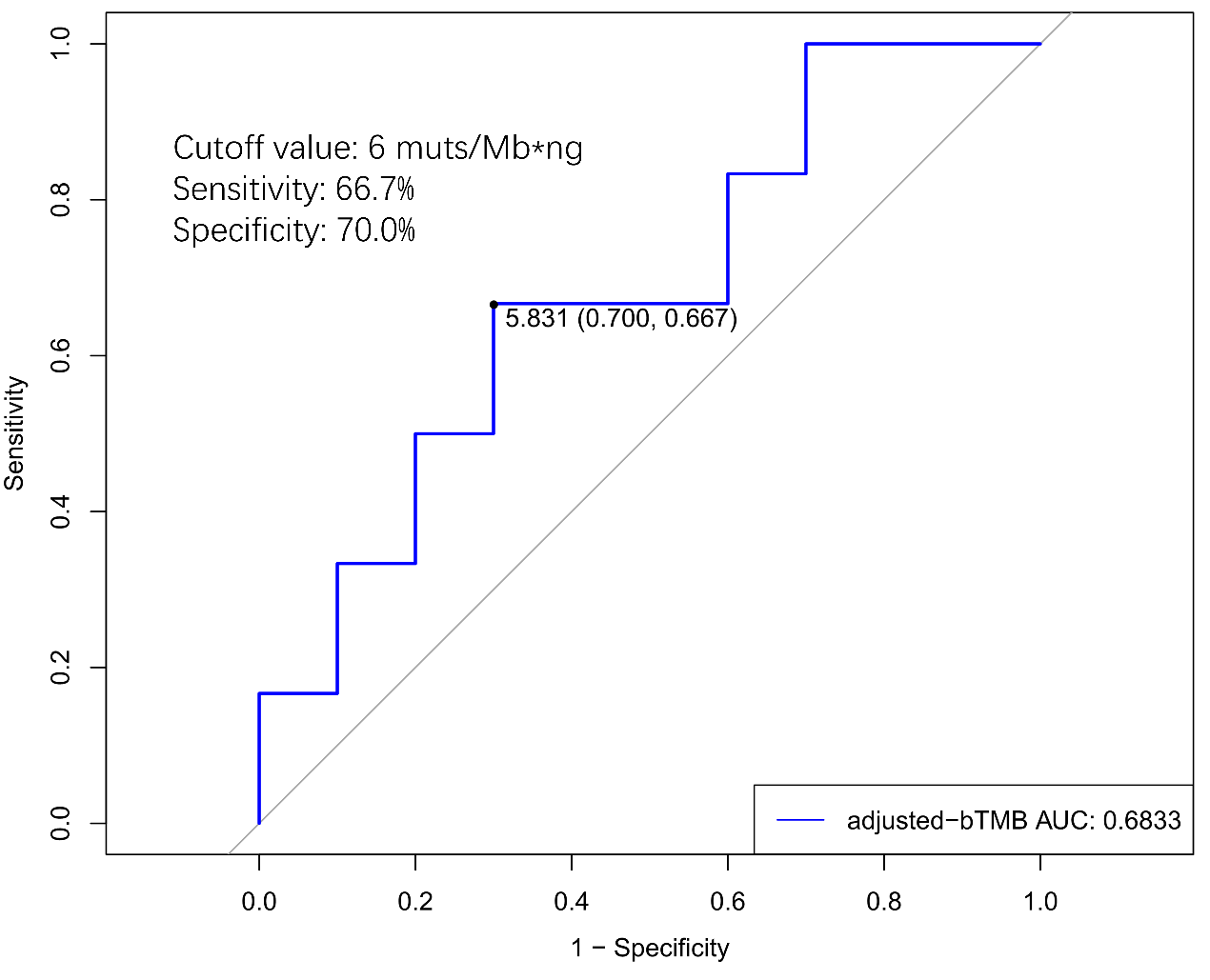


**Figure S7.** ROC curve of ctDNA adjusted bTMB to predict DCB in Shanghai and Wuhan (SH&WH) cohort.


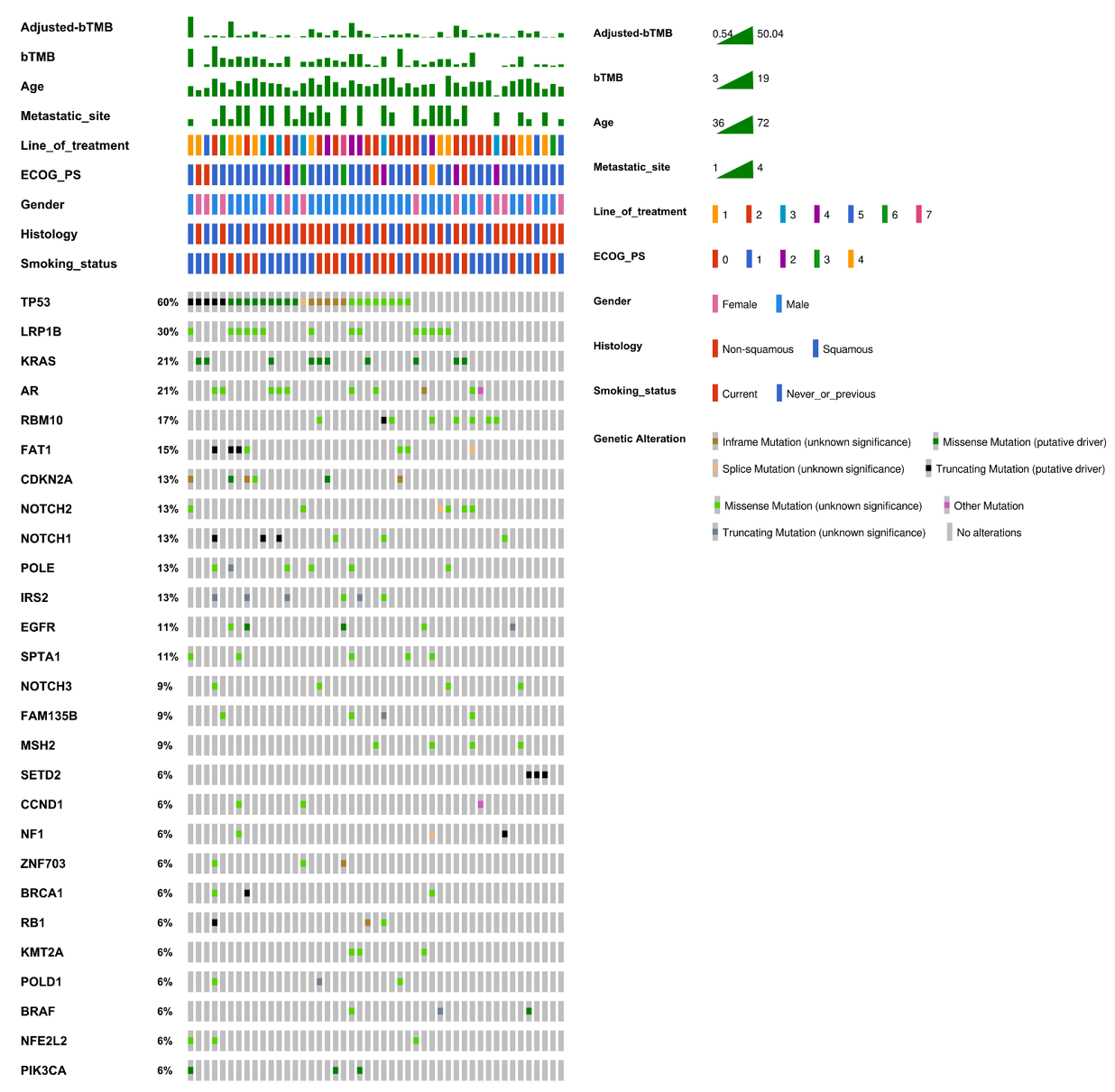


**Figure S8.** Oncoprint and clinical characteristics for patients of National Cancer Center (NCC) cohort.


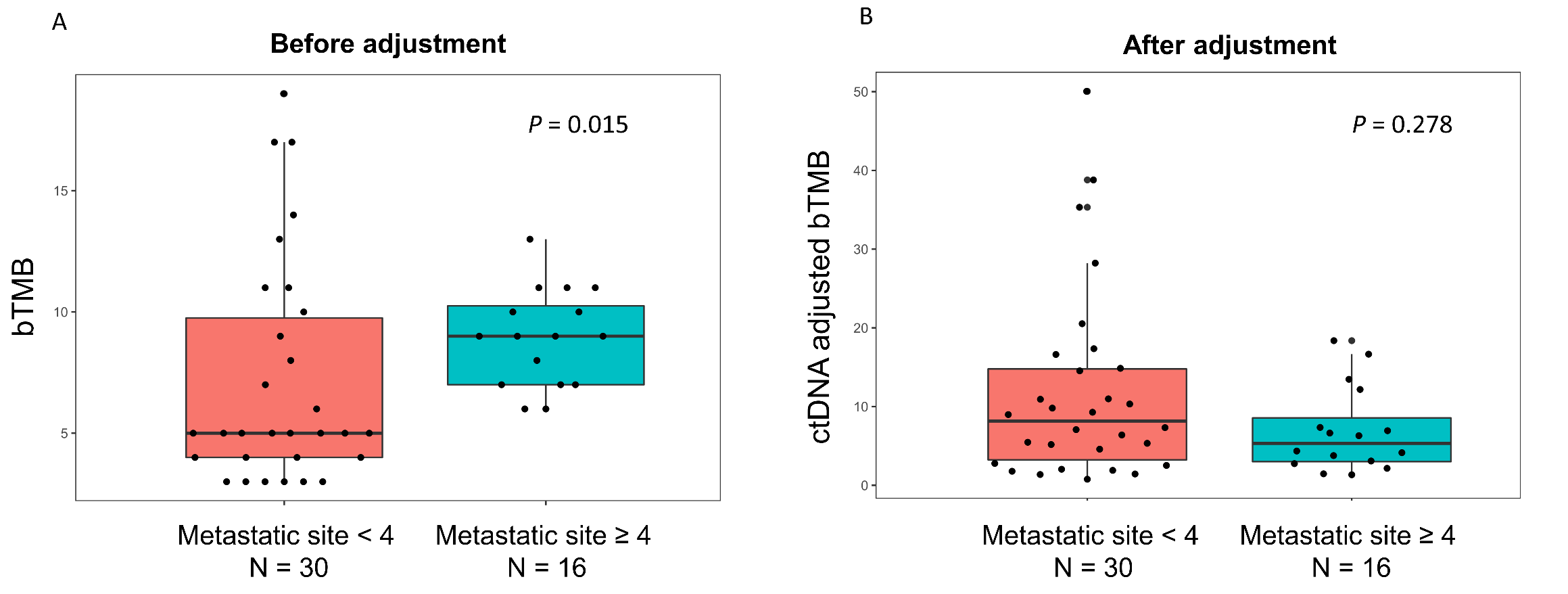


**Figure S9.** Comparisons of bTMB (A) and ctDNA adjusted bTMB (B) between patients with metastatic site < 4 and metastatic site ≥ 4.


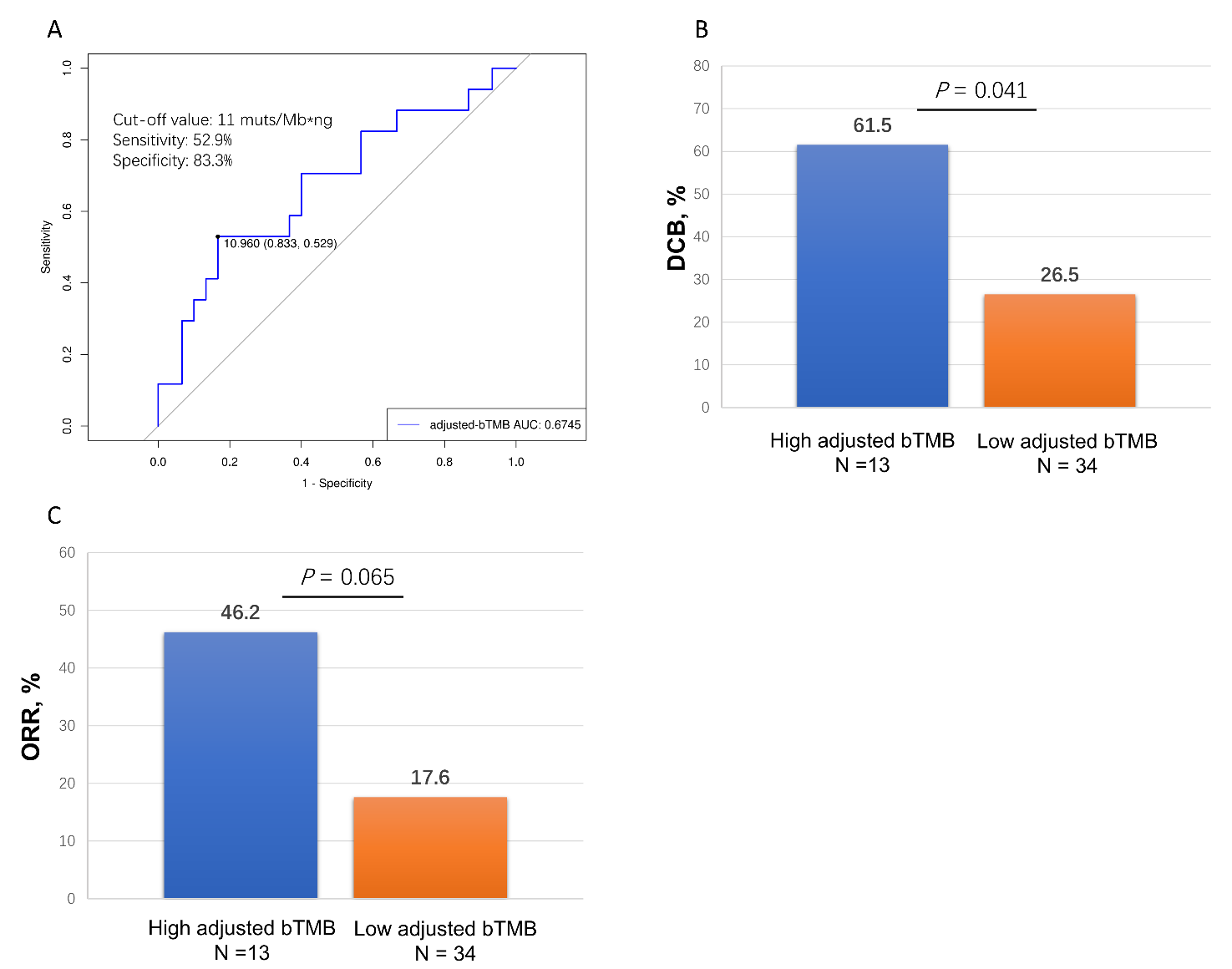


**Figure S10.** (A) ROC curve of ctDNA adjusted bTMB to predict DCB in National Cancer Center (NCC) cohort. Comparisons of (B) DCB and (C) ORR between patients with high and low ctDNA adjusted bTMB.


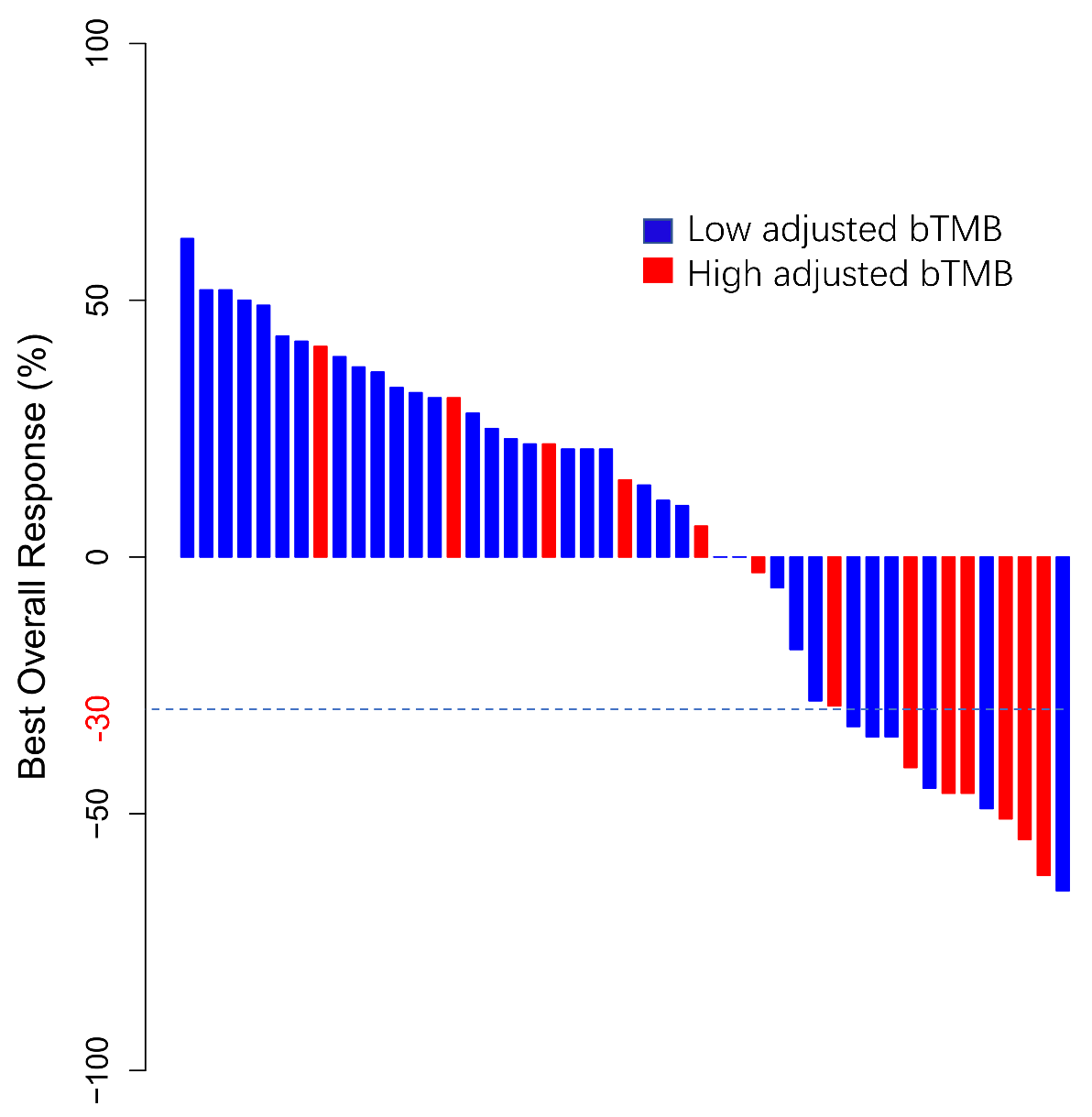


**Figure S11.** Waterfall plot of observed best response from anti–programmed cell death 1 (anti–PD-1) and anti–programmed cell death ligand 1 (anti–PD-L1) checkpoint inhibitors
